# Effects of Microcystin-LR Exposure on Spermiogenesis in Nematode *Caenorhabditis* *elegans*

**DOI:** 10.3390/ijms160922927

**Published:** 2015-09-22

**Authors:** Yunhui Li, Minhui Zhang, Pan Chen, Ran Liu, Geyu Liang, Lihong Yin, Yuepu Pu

**Affiliations:** 1 Key Laboratory of Environmental Medicine Engineering Ministry of Education, School of Public Health, Southeast University, Nanjing 210009, China; E-Mails: zhangminhui1990@163.com (M.Z.); ranliu@seu.edu.cn (R.L.); lianggeyu@163.com (G.L.); lhyin@seu.edu.cn (L.Y.); yppu@seu.edu.cn (Y.P.); 2 Department of Molecular Pharmacology, Albert Einstein College of Medicine, Bronx, NY 10461, USA; E-Mail: pan.chen@einstein.yu.edu

**Keywords:** *Caenorhabditiselegans*, microcystin-LR, spermiogenesis, reproductive toxicity

## Abstract

Little is known about the effect on spermiogenesis induced by microcystin-leucine arginine (MC-LR), even though such data are very important to better elucidate reproductive health. In the current work, with the aid of nematode *Caenorhabditis* *elegans* (*C. elegans*) as an animal model, we investigated the defects on spermiogenesis induced by MC-LR. Our results showed that MC-LR exposure induced sperm morphology abnormality and caused severe defects of sperm activation, trans-activation, sperm behavior and competition. Additionally, the expression levels of *spe-15* were significantly decreased in *C. elegans* exposed to MC-LR lower than 16.0 μg/L, while the expression levels of *spe-10* and *fer-1* could be significantly lowered in *C. elegans* even exposed to 1.0 μg/L of MC-LR. Therefore, the present study reveals that MC-LR can induce adverse effects on spermiogenesis, and those defects of sperm functions may be induced by the decreases of *spe-10*, *spe-15* and *fer-1* gene expressions in *C. elegans*.

## 1. Introduction

Harmful blooms of cyanobacteria occur in eutrophicated aquatic environments around worldwide and can produce a variety of toxins, including microcystins (MCs). MCs are a family of cyclic heptapeptides posing a public health threat to animals and human, among which microcystin-leucine arginine (MC-LR) is the most common and abundant variant [1,2]. MC-LR causes multiple lesions, including hepatotoxicity, neurotoxicity and kidney impairment [3,4]. In recent work, MC-LR has been transported rapidly to and accumulated in the gonads of mammals [5]. Animals treated with MC-LR show reduced sperm motility and concentration, and atrophy and obstruction of seminiferous tubules [6]. In addition, MC-LR exposure causes lesions of testicular Leydig cells [6], Sertoli cells [7], spermatogonia and spermatogenic cells [8]. Particularly, recent studies reported that spermatogenesis appeared to be disturbed [9] and various genes expressions associated with spermatogenesis were significantly altered [10] after MC-LR exposure. However, little information is available concerning the potential toxicity of MC-LR for male spermiogenesis.

Spermiogenesis is a complex, highly organized and regulated process. Round haploid spermatids undergo a series of morphological changes to transform into activated, mature and motility spermatozoa, which plays a very important role in the sperm transportation [11]. Errors in spermiogenesis may result in sperm abnormality that contributes to infertility [12]. However, it is difficult to detect the sperm defects stemming from impaired spermiogenesis in mammalian species [13,14].

Nematode *Caenorhabditis* *elegans* (*C. elegans*) has been widely used as a model animal for toxicity assessment and toxicological study [15,16]. *C. elegans* has a series of features and advantages that make it a powerful tool to illuminate and study the mechanisms of reproductive toxicity of chemicals. First, *C. elegans* is endowed with a highly differentiated and simple reproductive system that possesses three major fundamental biological processes of gametogenesis, including mitosis, meiosis and spermiogenesis [17,18]. Second, 60%–80% of human genes are homologous with those of *C. elegans*, including many of conserved reproductive-related genes [19]. Third, compared with the mammalian models, *in vitro* spermiogenesis can be completed easily in *C. elegans* [20]. Fourth, *C. elegans* is transparent, which provides clear observation of germ cells in mature and developing nematodes [21]. Additionally, our previous study has showed that *C. elegans* is useful for assessment of reproductive toxicity relating to gametogenesis [22]. In particular, it is reported that the *C. elegans* assay can provide a comprehensive screening platform for assessing chemical disruption of germline function [23]. Also, *C. elegans* has been used as an excellent model to elucidate the germline apoptosis caused by exposure to MC-LR [24].

In this study, to assess further the MC-LR-induced toxic effects on sperm morphology and function, we utilized *C. elegans* as an animal model to explore the severe defects and the potential toxic mechanism on spermiogenesis induced by MC-LR, with the aid of sperm activation, trans-activation, and sperm behavior and competition.

## 2. Results and Discussion

**Figure 1:**
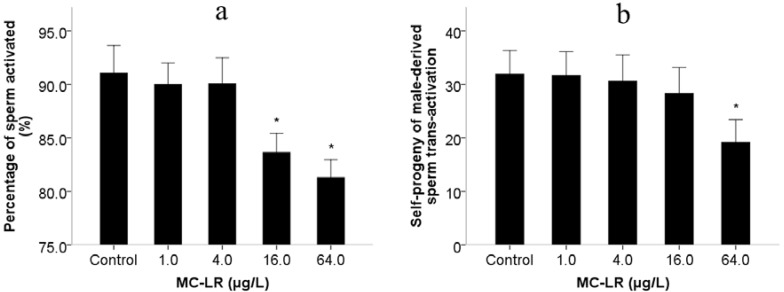
Effects of microcystin-leucine arginine (MC-LR) exposure on nematodes sperm activation (**a**) *him-5* sperm activation *in vitro* with Pronase; and (**b**) Number of self-progeny of male-derived sperm trans-activation. Bars represent means ± SEM. * *p* < 0.05 *vs*. the control group.

**Figure 2:**
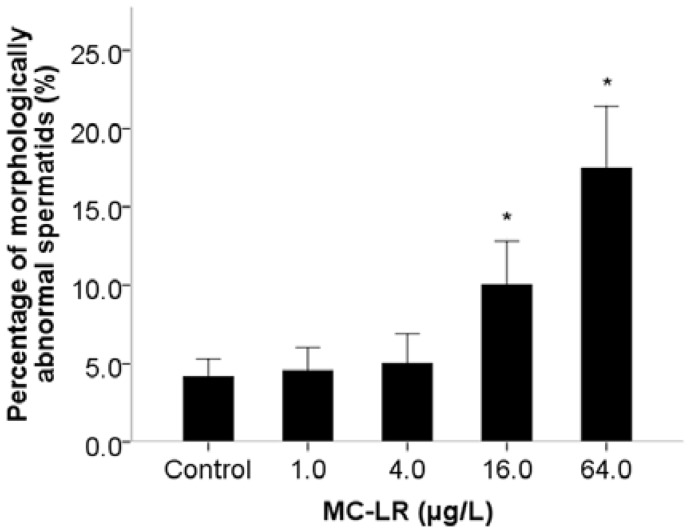
Effects of MC-LR exposure on spermatids morphology. Bars represent means ± SEM. * *p* < 0.05 *vs*. the control group.

**Figure 3:**
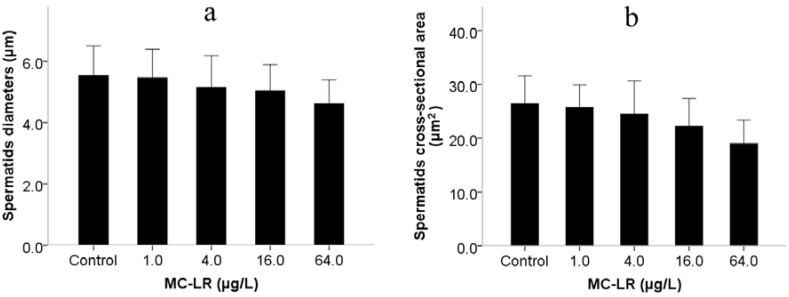
Effects of MC-LR exposure on spermatids size. (**a**) Spermatids diameters; and (**b**) Spermatids cross-sectional area. One male was dissected and 200 spermatids were analyzed. Bars represent means ± SEM.

Mammalian assays have indicated that MC-LR induces reproductive toxicity, although there are few reports on the effects of MC-LR exposure on spermiogenesis. This is to a large extent due to technical challenges in the process of spermatogenesis in mammalian assays, as well as *in vitro* tests [14]. Similar to the mammals, *C. elegans* has the conservative spermiogenesis to produce motile spermatozoons with pseudopodium instead of flagella. Sperm motility in *C. elegans* relies on pseudopod protrusion and retraction when hybridization occurs [25]. During the period of pseudopod extension in spermiogenesis, *C. elegans* spermatids can be activated *in vitro* by ionophore monensin, triethanolamine and Pronase [26], suggesting that activation is controlled by extracellular signals. Male-derived sperm activators are also involved in the process of pseudopod extension in spermiogenesis [27]. Therefore, we investigated pseudopod extension controlled by extracellular signals, activators of male-derived sperm determined by assays of sperm activation and trans-activation in spermiogenesis. Exposure to MC-LR increased the number of abnormal activated sperm with short pseudopods and reduced the percentage of activated sperm (Figure 1a). The inhibition of sperm activation suggested that MC-LR might damage the sperm extracellular signals. Sperm trans-activation was also performed to observe that male-derived activators transactivated hermaphrodite-derived sperm to produce hermaphrodite-derived progeny (self-progeny). The result showed that the number of self-progeny in *C. elegans* was decreased when exposed to 64.0 μg/L (Figure 1b). The decline of sperm transactivation suggested that MC-LR might damage male-derived sperm activators. We further found that spermatids morphology was significantly altered following MC-LR exposure (Figure 2), while spermatids diameter and cross-sectional area in *C. elegans* showed nonsignificant changes compared with the control groups (Figure 3). Therefore, we suspected that MC-LR suppressed sperm activation and transactivation, which may result directly in abnormalities in spermatids morphology and pseudopod extension. This suggests that these defects in *C. elegans* exposed to MC-LR may be largely related to spermiogenesis.

**Figure 4:**
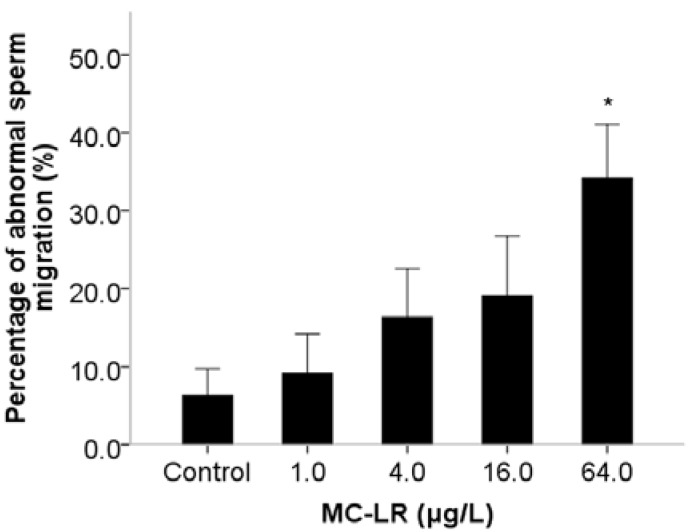
Effects of MC-LR exposure on sperm migration. The number of abnormal sperm migration worms and total worms were counted. Bars represent means ± SEM. * *p* < 0.05 *vs*. the control group.

**Figure 5:**
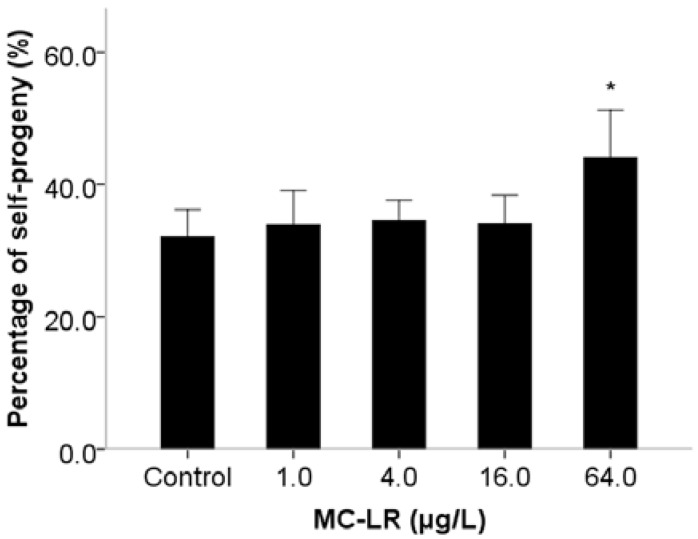
Effects of MC-LR exposure on sperm competitiveness. The number of self-progeny and outcrossed-progeny were counted. Bars represent means ± SEM. * *p* < 0.05 *vs*. the control group.

During the period of male mated with female, male-derived sperm are activated after ejaculation into the female reproductive tract and targeted for spermathecae. MitoTracker Red (Invitrogen, Carlsbad, CA, USA) was used to visualize male-derived sperm migration in female reproductive tract. It suggested that sperm migration is suppressed if the mitotracker-labeled sperms are seen in ectopic positions such as uterus and vulva in female [27,28]. Our results showed that sperm migration of *C. elegans* was suppressed by exposure to MC-LR at a dose of 64.0 µg/L (Figure 4). Further, we also investigated the ability of male-derived sperm competition through accounting the numbers of self progeny and outcross progeny. Compared with the 33% self-progeny percentage in the control group, the percentage with 64.0 µg/L MC-LR was significantly increased to 43% (*p* < 0.05) (Figure 5). Previous studies indicated that sperm competitiveness was strongly correlated with sperm size [29], while our results showed no change in spermatids diameters and cross-sectional area. This implies that MC-LR might inhibit sperm competitiveness by other unidentified mechanisms. We used *C. elegans* as a model to bring new insights into the reproductive effects of MC-LR by providing the first evidence that MC-LR exposure can result in defects of spermiogenesis, including extracellular signals and auto-activators of sperm activation, sperm behavior and competitiveness.

**Figure 6:**
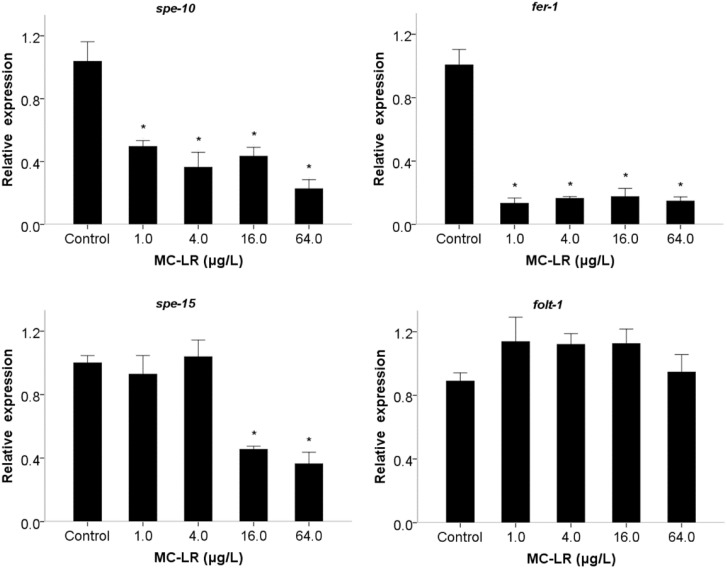
Effects of MC-LR exposure on relative mRNA expression levels of genes involved in spermiogenesis. Bars represent means ± SEM. * *p* < 0.01 *vs*. the control group.

Based on the mentioned impairments of MC-LR, the expression levels of four genes (*spe-10*, *spe-15*, *fer-1* and *folt-1*) were analyzed to explore the potential molecular mechanisms. *spe-10* is required for spermatids development in spermatogenesis and fertility [30], while *spe-15* is related to spermiogenesis or sperm activation [31]. *fer-1* plays a role in sperm pseudopods extension and motility [32]. *folt-1* is related to germline function and sperm count [33,34]. Besides, *spe-10*, *spe-15* and *fer-1* are involved in the transportation of sperm fibrous body-membranous organelles (FB-MOs) and FBs disassemble, respectively. Therefore, the three genes were selected based on the continuous development of FB-MOs, which is important for spermatogenesis [18]. We speculated that MC-LR might damage spermiogenesis by disrupting the development of FB-MOs, although the more serious decrease in expression of three genes was not completely consistent with the toxic changes of spermiogenesis (Figure 6). A particularly interesting feature is that we did not observe any change in *folt-1* expression level (Figure 6), although MC-LR decreased sperm numbers in *C. elegans* (data not shown). Therefore, the results imply that *folt-1* is not involved in the regulation of MC-LR-induced spermiogenesis defects. Other genes related to signaling pathways require further exploration.

A previous study reported that spermatogenesis is disturbed by exposure to MC-LR [9]. Here, we extended this observation by providing evidence that MC-LR induced spermiogenesis defects and subsequent process abnormality until fertilization of *C. elegans*. Our results also show that *C. elegans*, as a simple and reliable model organism, has a unique advantage in studying the process of spermatogenesis and may complement *in vitro* and whole-organism assays in reproductive toxicology.

## 3. Experimental Section

The strains used in this study were wild-type N2, *him-5(e1490)*, *fog-2(q71)*, *dpy-5(e61)*, *fer-1(hc1)*; *him-5(e1490)* and *spe-8(hc40)*; *dpy-4(e1166)*, originally obtained from the Caenorhabditis Genetics Center (CGC, University of Minnesota, Minneapolis, MN, USA). Nematodes were grown and maintained at 20 °C as described [21]. Gravid nematodes were removed OP50 by M9 buffer and eggs were harvested by a bleaching mixture (0.45 M NaOH, 2% HOCl). Age-synchronized populations of L4-larvae nematodes were obtained by the collection cultured in 20 °C.

MC-LR (purity ≥ 95%) was purchased from Alexis Biochemicals Corporation (Lausen, Switzerland). MC-LR (1 mg) was dissolved in 1 mL of methanol and diluted to 1000 µg/L with M9 buffer to prepare the stock solution. The concentrations of MC-LR of working solutions were analyzed by Agilent 1100 liquid chromatograph (Agilent Technologies, Santa Clara, CA, USA). The HPLC conditions were performed in our previous study as description [35]. MC-LR exposure was performed according to the agar toxicity test as described [36]. The final MC-LR concentrations of culture medium were 1.0, 4.0, 16.0 and 64.0 µg/L, respectively. The nematodes were exposed to MC-LR for 48-h at 20 °C and the solvent controls were prepared by the same way.

The sperm activation was performed according to previous description [37]. *him-5* male was dissected to release sperm into SM buffer containing 10 μL Pronase E (200 μg/mL). The percentage of activated sperm was calculated as follows: activated sperm/total sperm × 100. 10 nematodes were used in control and exposed groups, and three replicates were performed.

Four virgin *fer-1*; *him-5* males were crossed at the ratio of 4:1 with one *spe-8*; *dpy-4* hermaphrodite to analyze the transactivation as described [38]. Each plate was considered a single mating trial, and ten plates were scored. Only mating successful hermaphrodites were transferred to the new NGM plate, respectively. Progeny were scored until the hermaphrodites were no eggs lying. Three replicates were performed.

The sperm size and morphology were measured and analyzed as previously published [22], *him-5* male was dissected to release spermatids. Five different visual fields about 200 spermatids for each sample were captured under a differential interference contrast (DIC) microscope (Olympus BX41, Olympus, Tokyo, Japan) [39]. The spermatids diameter, cross-sectional area and morphology were measured and analyzed using soft image-pro plus 6.0 (Media Cybernetics, Rockville, MD, USA). 10 nematodes were used and three replicates were performed.

To analyze the mitotracker sperm migration, observation of the mitotracker-labeled male-derived sperms movement within female reproductive tracts was based on previous description [28]. Synchronized *him-5* males were labeled and incubated in both MitoTracker Red CMXRos (Invitrogen, Carlsbad, CA, USA) and MC-LR solution for 48-h at 20 °C, then crossed at the ratio of 3:1 with young adult of *fog-2* female for 8-h. Then female was transferred to a new NGM plate for 2-h ensuring enough time for sperms migrating to the spermatheca before the microscopy observation. Mitotracker fluorescence was observed by fluorescence microscopy (Olympus FSX100, Olympus, Tokyo, Japan). Only mating successful and corpse integrity worms were practical. The abnormal sperms migration were seen in ectopic positions such as uterus and vulva in female. The percentage of abnormal sperm migration (abnormal migration worms/total worms × 100) was calculated. 10 nematodes were used and three replicates were performed.

The sperm competition was performed as previously described [40]. L4 *him-5* males were exposed to MC-LR for 48-h, then crossed with *dpy-5* hermaphrodites (4:1) for 24-h at 20 °C. Ten successful mating trails were tested. The number of short-limb phenotype (self-progeny) and normal-limb phenotype (outcrossed-progeny) were counted. Three replicates were performed.

To determine *C. elegans* spermiogenesis relative genes expression levels, real-time Quantitative PCR (qRT-PCR) was performed to measure the reverse transcription products using SYBR Green I dye (TOYOBO, Osaka, Japan). The primers were performed in Table 1. Relative expression levels were determined with Mastercycler gradient PCR (Eppendorf, Hamburg, Germany) and ABI 7300 Quantitative PCR (ABI, Carlsbad, CA, USA). Relative RNA expression levels were determined using the 2^−ΔΔ*C*t^ method with three replicates for each group [22].

**Table 1:** Gene primers tested in the study.

Gene Primer		Sequence	Annealing Temperature
*spe-10*	Forward	TTTTATTGTCGGCGGAGTGT	57.8 °C
	Reverse	CGATGACTGCGAACTTTGAG	
*spe-15*	Forward	GGAGTTTTGGATGTCGCTGGTT	60.4 °C
	Reverse	GCTCTCTGGGTGAAATGTTGGA	
*fer-1*	Forward	AATGGATGGAATGCTGTTGGTC	57.8 °C
	Reverse	AACGCTTTCTGAAGTTGTGGTG	
*folt-1*	Forward	TCCATTCCTCACTCCGTTTCTA	60.4 °C
	Reverse	GCATCTGCCATACTCCTTTACC	
*act-1*	Forward	ATGTGTGACGACGAGGTT	60.4 °C
	Reverse	GAAGCACTTGCGGTGAAC	

Data were plotted as means ± Standard Error of the Mean (SEM). One-way ANOVA/Dunnett’s *t*-test was used for comparison between the control and the exposed groups. Probability levels of 0.05 and 0.01 were considered statistically significant. Statistical analysis was processed using SPSS 13.0 (SPSS Inc., Chicago, IL, USA).

## 4. Conclusions

In our current works, we found that *spe-10*, *spe-15*, and *fer-1* gene are involved in mediating MC-LR-induced sperm extracellular signals and sperm own activators abnormalities, and this could result in sperm abnormal pseudopods. Those abnormalities were effected together to induce sperm morphological defects of the activation process in spermiogenesis and further inhibit sperm migration, eventually lead to sperm competitiveness decline. Although the study showed a potential signal transduction pathway in modulating MC-LR-induced spermiogenesis abnormality, more related mechanisms should be further explored.
